# Impact of Direct-Acting Antiviral Therapy on Liver Fibrosis Regression among People with Chronic HCV Infection: Results from a Real-Life Cohort in Patients Who Achieved Sustained Virological Response

**DOI:** 10.3390/medicina59040814

**Published:** 2023-04-21

**Authors:** Alejandro García-Ros, Senador Morán, Virginia Núñez, Gonzalo García-Ros, Guadalupe Ruiz, José García-Solano

**Affiliations:** 1 Department of Emergency Medicine, Santa Lucía University Hospital, 30202 Cartagena, Spain; 2 Department of Gastroenterology and Hepatology, Santa Lucía University Hospital, 30202 Cartagena, Spain; 3 Department of Psychiatry, Mental Health Center, 30201 Cartagena, Spain; 4 Mining and Civil Engineering Department, Polytechnic University of Cartagena, 30202 Cartagena, Spain; gonzalo.garcia@upct.es; 5 Foundation for Health Training and Research of the Region of Murcia, 30120 Murcia, Spain; 6 Department of Pathological Anatomy, Santa Lucía University Hospital, 30202 Cartagena, Spain

**Keywords:** chronic hepatitis C virus infection, direct-acting antiviral therapy, sustained virological response, liver fibrosis regression

## Abstract

*Background and Objectives*: The global prevalence of chronic hepatitis C virus (HCV) infection is 0.8%, affecting around 58 million people worldwide. Treatment with DAAs reduces all-cause HCV mortality by 49–68%. This work aims to determine whether there is liver fibrosis regression (LFR) in patients who achieved Sustained Virological Response (SVR) after treatment with DAAs. *Materials and Methods*: An analytical, observational, single-center, and cohort study was carried out. The final sample consisted of 248 HCV-infected patients. All started treatment with DAAs between January 2015 and December 2017. Five measurements were performed to determine the fibrotic stage in patients (measured in kilopascals (kPa)) using transient elastography (FibroScan^®^, Echosens, The Netherlands). *Results*: Taking the baseline fibrotic stage as a reference, the distribution in subgroups was as follows: 77 F4 patients (31.0%); 55 F3 patients (22.2%); 53 F2 patients (21.4%); and 63 F0/F1 patients (25.4%). There were 40 patients (16.1%) with at least one HCV complication and 13 (5.2%) who developed hepatocellular carcinoma. The overall LFR rate was 77.8% (144 of 185 F2/F3/F4 patients, *p* = 0.01) at the end of the follow-up period. The highest mean FibroScan^®^ values were observed in patients with: “male gender”; “metabolic syndrome”; “subtype 1a”; “NRP DAA”; “at least one HCV complication”; “death from HCV complications”; and “liver transplantation requirement”. *Conclusions*: Treatment with DAAs achieved high rates of LFR and a decrease in mean FibroScan^®^ values in all subgroups.

## 1. Introduction

According to World Health Organization (WHO) estimates published in the 2021 Global Progress Report, the global prevalence of hepatitis C virus (HCV) infection is 0.8% [1]. This is a serious public health problem, with approximately 58 million people worldwide suffering from chronic HCV infection. So early diagnosis and treatment are essential to reduce morbidity and mortality [2,3]. Eight clinically relevant genotypic variants and a total of 93 HCV subtypes have been described, each viral genotype (GT) having a different therapeutic response. The most frequent is GT 1, with subtype 1b the most relatively prevalent [4]. Injecting drugs is the most common way of acquiring HCV mono-infection [5].

Around 30–35% of HCV-infected patients achieve spontaneous virus clearance in about six months. Then, two out of three patients will develop chronic HCV infection. Liver fibrosis often occurs as a result of long-standing chronic HCV infection. It is estimated that progression to cirrhosis is 16% at 20 years and 41% at 30 years [6,7]. In addition, HCV complications (i.e., ascites, encephalopathy), diabetes mellitus, obesity, and high active alcohol use are associated with advanced fibrotic stages [8]. The most relevant complication is hepatocellular carcinoma, whose annual incidence is close to 3% [9].

HCV-infected patients with advanced fibrotic stages have a 10% annual risk of progression to cirrhosis. When it appears, the annual risk of decompensation is close to 15–20%, and the annual incidence of hepatocellular carcinoma rises to 8–10% [7,10]. The Child–Pugh scale evaluates mortality in cirrhotic patients, which is 55% at one year and 65% at two years in Child–Pugh class C patients [11]. Therefore, achieving liver fibrosis regression (LFR) is essential to reduce the morbidity and mortality caused by HCV complications [12,13].

The goal of treating chronic HCV infection is to achieve a Sustained Virological Response (SVR), defined as an undetectable viral load 12 weeks after completing treatment. If this is achieved, progression to cirrhosis, development of hepatocellular carcinoma, and mortality will be reduced [14,15]. The first drug used to treat chronic HCV infection was Interferon (IFN) monotherapy, with little success [16]. 

Compared to former Interferon-based therapeutic regimens, Direct-acting antiviral (DAA) therapeutic regimens have shown higher rates of SVR (90–95% vs. 50–70%) [17]. In addition, treatment with DAAs reduces all-cause HCV mortality by 49–68% [18]. Pangenotypic therapeutic regimens have been shown to be highly effective in all GTs [19]. Due to their therapeutic success and availability, the WHO has recommended their use for all adults with chronic HCV infection [1]. Since it is possible to modify the natural history of chronic HCV infection, the high price of DAAs could be offset by savings in healthcare costs [20].

DAAs have the following advantages: easier dosage; shorter duration (12 weeks in most cases); and better tolerance (few side effects). At least two drugs must be combined, each from a different family. According to their therapeutic target, three families are distinguished: NS3/4A protease inhibitors; NS5A protein inhibitors; and NS5B polymerase inhibitors [21].

Traditionally, liver biopsies have been the gold standard to determine the condition of liver parenchyma [22]. In recent years, other non-invasive methods have been used to assess liver architecture. Transient elastography (FibroScan^®^) emits low-frequency waves throughout the liver, allowing specialists to study their propagation. Using this technique, the fibrotic stage can be estimated quite accurately [23,24].

Many studies have evaluated whether LFR occurs in patients who achieved SVR after treatment with IFN therapeutic regimens. This work aims to determine whether there is LFR in patients who achieved SVR after treatment with DAAs since similar studies are scarce due to the recent appearance of DAAs.

## 2. Materials and Methods

### 2.1. Study Sample

**Figure 1:**
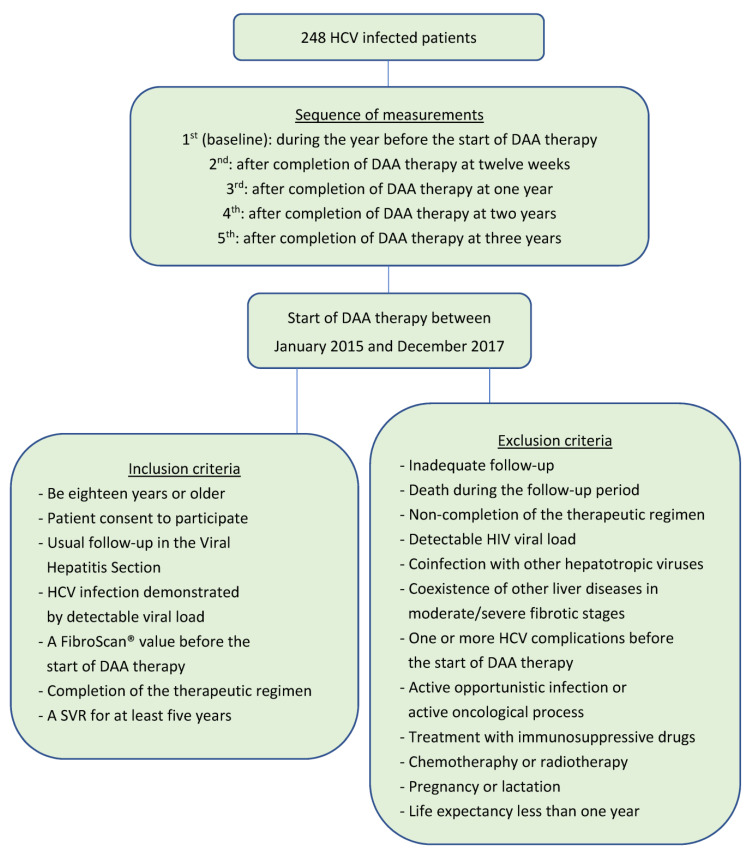
Study design (participants were enrolled until 31 December 2017 and were followed up until 31 December 2020). DAA, direct-acting antiviral; HCV, hepatitis C virus; HIV, human immunodeficiency virus; SVR, sustained virological response.

Figure 1 shows the study design, which consisted of an analytical, observational, single-center, and cohort study. Patients from a retrospective cohort of people with chronic HCV infection were included using simple random sampling. The final sample consisted of 248 HCV-infected patients. All started treatment with DAAs between January 2015 and December 2017 and were regularly followed up in the Viral Hepatitis Section of the Hepatology Area in Santa Lucía University Hospital (Cartagena, Spain). Simultaneously, patients coinfected with the human immunodeficiency virus (HIV) were treated and periodically followed up in the Infectious Diseases Section of the Internal Medicine Area in the same hospital. In order to avoid alteration of FibroScan^®^ values, only HCV/HIV coinfected patients with an undetectable HIV viral load were included.

A total of 42 people were excluded during the follow-up period, in most cases (27, 64.3%) due to inadequate follow-up, defined as non-attendance to at least one measurement after finishing treatment. Other causes were death during the follow-up period (3, 7.1%), non-completion of DAA therapy (2, 4.8%), coinfection with other hepatotropic viruses (3, 7.1%), the coexistence of other liver diseases in moderate/severe fibrotic stages (1, 2.4%), one or more HCV complications before the start of DAA therapy (5, 11.9%), and treatment with immunosuppressive drugs (1, 2.4%).

In order to evaluate how the FibroScan^®^ value changed after the hepatitis C cure, only those patients who achieved SVR after the completion of the DAA therapy were finally included in the study [14]. The choice of the DAA therapeutic regimen was based on the recommendations issued by the “Spanish Association for the Study of the Liver (AEEH)” [25]. Then, all patients were assigned a personalized therapeutic regimen according to their GT and baseline fibrotic stage. Moreover, a pretreatment resistance analysis was performed in patients who had previous DAA therapy to determine if there were resistance-associated substitutions (RASs) to DAAs. If they appeared, the therapeutic regimen was chosen based on it [21].

The incidence of the following HCV complications was studied: “hepatocellular carcinoma”; “ascites”; “varicose veins”; “encephalopathy”; “kidney failure”; and “liver transplantation requirement”. In addition, the incidence of patients with “at least one HCV complication” and “death from HCV complications” was also analyzed.

Based on treatment history for chronic HCV infection, there were 6 types of patients:

(1) Treatment-naïve (NAÏVE): patients who had not previously received treatment for chronic HCV infection.
(2) Poor tolerance to a previous IFN therapy (PTP IFN): patients who did not complete a previous IFN therapy due to severe side effects.
(3) Partial response to a previous DAA therapy (PRP DAA): patients with new-onset detectable viral load after achieving SVR with a previous DAA therapy.
(4) No response to a previous DAA therapy (NRP DAA): patients who did not achieve SVR after completing a previous DAA therapy.
(5) Partial response to a previous IFN therapy (PRP IFN): patients with new-onset detectable viral load after achieving SVR with a previous IFN therapy.
(6) No response to a previous IFN therapy (NRP IFN): patients who did not achieve SVR after completing a previous IFN therapy.

### 2.2. FibroScan^®^

FibroScan^®^ is a non-invasive, fast, and reliable technique with good intraobserver reproducibility and a wide range of values (2.5–75 kPa). The patient must be in the supine position, with the right arm behind the neck. Measurements are taken with a gel transducer placed on the intercostal skin surface of the right hepatic lobe. This technique has limitations in patients with breach of fast, alcohol abuse, obesity, reduced intercostal spaces, ascites, acute liver failure, extrahepatic cholestasis, and congestive heart failure.

FibroScan^®^ interpretation was carried out in accordance with standard clinical practice, following an identical action protocol by the same two doctors throughout the follow-up period. Ten measurements were performed, taking the median in kPa as the result. IQR/median < 20% was used as the validity criterion of the measurement. Abdominal ultrasound was previously performed in patients with ascites, with FibroScan^®^ measurements being performed only if mild ascites had been observed [23,24].

Five measurements were performed to determine the fibrotic stage in patients (measured in kilopascals (kPa)) using FibroScan^®^. The sequence of measurements was 1st (baseline) during the year before the start of DAA therapy, with the remaining four measurements performed after the completion of DAA therapy at twelve weeks (2nd), at one year (3rd), at two years (4th), and three years (5th), respectively.

The METAVIR scale was designed to assess liver architecture, grading liver fibrosis in the same stages as the histological analysis [26]. As shown in Table 1, this scale was used to distribute patients into four subgroups according to their baseline measurement: F0/F1 subgroup (≤7.4 kPa), F2 subgroup (7.5–9.4 kPa), F3 subgroup (9.5–12.4 kPa), and F4 subgroup (≥12.5 kPa).

The impact of SVR was evaluated in terms of LFR, defined as a decrease in at least one fibrotic stage on the METAVIR scale compared to the baseline measurement at the end of the follow-up period [12]. This could not be applied to the F0/F1 subgroup, as they had the lowest fibrotic stage.

In order to show the impact of SVR in F0/F1 patients and F4 patients who did not achieve LFR, the term “decreasing trend in FibroScan^®^ value” was arbitrarily proposed. This term is not based on current scientific evidence. It refers to a quantitative reduction in FibroScan^®^ value of at least 10% compared to the baseline measurement at the end of the follow-up period.

### 2.3. HCV Viral Load and GT

Two frozen plasma standards were needed to determine viral load and one for genotyping. Each standard contained 1.5 mL of patient plasma.

Viral load was measured three times: before the start of treatment; in the middle of treatment; and 12 weeks after its completion to determine SVR. The GT was also targeted in the first measurement.

### 2.4. Statistical Analysis

Qualitative variables were described in frequency and percentage. The χ2 test or Fisher exact test was used to compare variables. The Kolmogorov–Smirnov test was used to determine quantitative variables with a normal distribution. Those with normal distribution were expressed as mean and standard deviation, using Student’s “*t*” for independent data (two groups) or ANOVA (more than two groups) to compare them. Those with a non-normal distribution were expressed in the median and interquartile range, using the Mann–Whitney U (two groups) or the Kruskal–Wallis H (more than two groups) to compare them with qualitative variables. The comparison between quantitative variables was carried out using simple logistic regression. Pearson’s test was used to study the association between linear correlations, and Kendall’s test was applied to nonlinear correlations.

Statistical significance was reached with a *p*-value < 0.05. The magnitude of the significance was quantified by a difference in means. Moreover, confidence intervals were used to increase the accuracy of the analysis.

## 3. Results

### 3.1. Baseline Characteristics

The prevalence of each baseline characteristic of patients is provided in Table 2. The age ranged from 28 to 83 years. Male predominance (175 of 248, 70.6%, *p* = 0.011) was observed.

Regarding comorbidities, there were 24 patients (9.7%, *p* = 0.007) with high active alcohol use, 33 (13.3%, *p* = 0.087) with mild liver steatosis, 24 (9.7%, *p* = 0.042) with metabolic syndrome, and 30 (12.1%, *p* = 0.065) with HIV infection.

Based on treatment history for chronic HCV infection, there were 159 (64.1%, *p* = 0.029) NAÏVE, 8 (3.2%) PTP IFN, 11 (4.5%) PRP DAA, 6 (2.4%) NRP DAA, 28 (11.3%) PRP IFN, and 36 (14.5%) NRP IFN.

Regarding genotypic information, GT 1 (188, 75.8%, *p* = 0.018) was the most frequent, with subtype 1b (108, 43.6%) having a higher relative prevalence than subtype 1a (80, 32.2%). GTs 2, 3, and 4 showed the following prevalence: 1.3% (3, *p* = 0.018); 14.5% (36); and 8.4% (21), respectively.

Taking the baseline fibrotic stage as a reference, the distribution in the subgroups was as follows: 77 F4 patients (31.0%); 55 F3 patients (22.2%); 53 F2 patients (21.4%); and 63 F0/F1 patients (25.4%).

### 3.2. DAA Therapy

There were therapeutic regimens of 8 weeks (13 patients, 5.2%), 12 weeks (185 patients, 74.6%), 16 weeks (7 patients, 2.8%), 24 weeks (38 patients, 15.4%), and 40 weeks (5 patients, 2.0%). “Ombitasvir/Paritaprevir/Ritonavir + Dasabuvir 12 weeks” (44 patients, 17.7%) was the most frequently chosen therapeutic regimen.

Of the 17 patients with previous DAA therapy, there were 5 (29.4%) with RASs to DAAs, 4 of them (1.6%) in the NS5A region and 1 (0.4%) in the NS3 region. The former had the A92E, L31M, Y93C, and Y93H polymorphisms; the latter had the Q80K polymorphism. This occurred in patients with subtype 1a (2, 0.8%), subtype 1b (2, 0.8%), and GT 3 (1, 0.4%).

There were no serious adverse drug reactions. Only eight patients (3.2%) had mild adverse drug reactions, so none interrupted treatment.

### 3.3. Complications

The incidence of each complication developed according to the baseline fibrotic stage is provided in Table 3. Overall, 40 patients (16.1%) had at least one HCV complication, and 23 (9.3%) had more than one. Then, there were 14 deaths (5.6%), and 5 of these 14 (35.7%) were from HCV complications.

There were 13 patients (5.2%) who developed hepatocellular carcinoma, 14 (5.6%) with ascites, 19 (7.8%) with gastric/esophageal varicose veins, 5 (2.0%) with encephalopathy, and 11 (4.4%) with kidney failure. In addition, there were 6 patients (2.4%) with liver transplantation requirement.

As Table 3 shows, most HCV complications appeared in F4 patients, with varicose veins having the highest incidence.

### 3.4. FibroScan^®^ Values

#### 3.4.1. Mean Values in Each Subgroup

**Figure 2:**
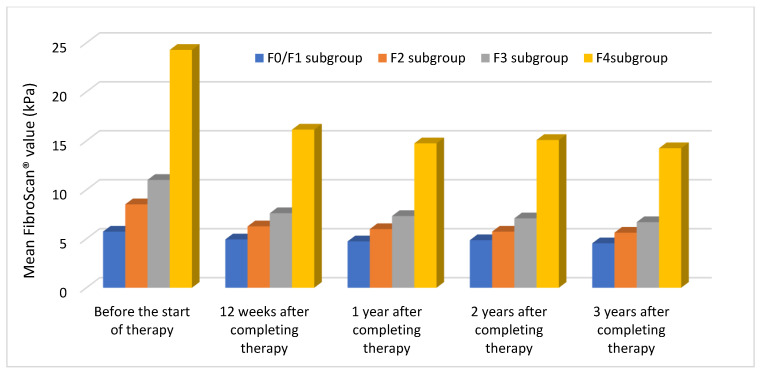
Trend of mean FibroScan^®^ values in each subgroup according to the baseline fibrotic stage throughout the follow-up period.

As shown in Figure 2, a decrease in mean values was observed in all subgroups at the end of the follow-up period.

F4 patients: 1st 24.282 kPa; 2nd 16.149 kPa; 3rd 14.742 kPa; 4th 15.087 kPa; and 5th 14.244 kPa.

F3 patients: 1st 11.007 kPa; 2nd 7.609 kPa; 3rd 7.322 kPa; 4th 7.087 kPa; and 5th 6.700 kPa.

F2 patients: 1st 8.517 kPa; 2nd 6.266 kPa; 3rd 5.989 kPa; 4th 5.726 kPa; and 5th 5.623 kPa.

F0/F1 patients: 1st 5.730 kPa; 2nd 4.933 kPa; 3rd 4.733 kPa; 4th 4.860 kPa; and 5th 4.533 kPa.

#### 3.4.2. Success Rates

The overall LFR rate was 77.8% (144 of 185 F2/F3/F4 patients, *p* = 0.01) at the end of the follow-up period. The success rate was 57.2% (44 of 77, *p* = 0.01) in F4 patients, 92.7% (51 of 55, *p* = 0.01) in F3 patients, and 92.4% (49 of 53, *p* = 0.01) in F2 patients.

Regarding F4 patients who did not achieve LFR, 72.3% (24 of 33, *p* = 0.01) decreased their mean values at the end of the follow-up period. Moreover, 60 of 63 F0/F1 patients (95.2%, *p* = 0.01) also decreased them.

In summary, patients in the F4 subgroup showed the lowest success rates, similar to the remaining subgroups.

#### 3.4.3. Patient Variables

This subsection describes and analyzes mean FibroScan^®^ values based on gender, comorbidities, GT, and treatment history for chronic HCV infection.

**Figure 3:**
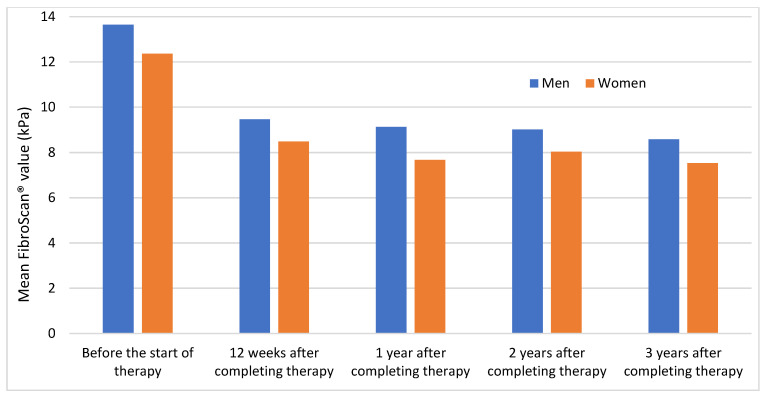
Trend of mean FibroScan^®^ values in men vs. women throughout the follow-up period.

As shown in Figure 3, the mean values were slightly higher in men than in women. Both genders showed a large decrease in their values at the second measurement and remained stable at subsequent measurements. Further information regarding these results can be found in Table S1 (Supplementary Materials).

**Figure 4:**
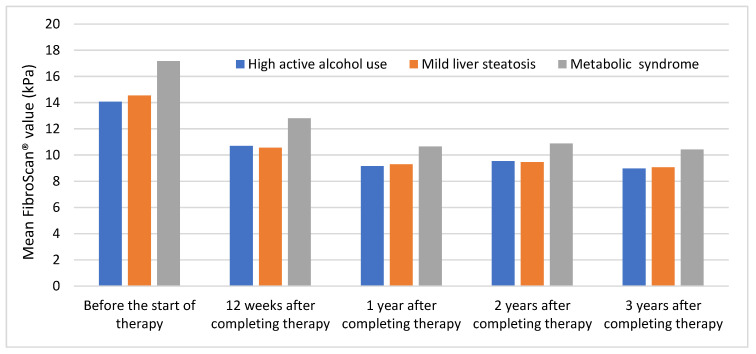
Trend of mean FibroScan^®^ values in patients with high active alcohol use, mild liver steatosis, and metabolic syndrome throughout the follow-up period.

As shown in Figure 4, the highest mean values were observed in patients with metabolic syndrome. The other subgroups showed identical values throughout the follow-up period.

All subgroups showed a large decrease in their values at the second measurement and remained stable at subsequent measurements. Further information regarding these results can be found in Table S2 (Supplementary Materials).

**Figure 5:**
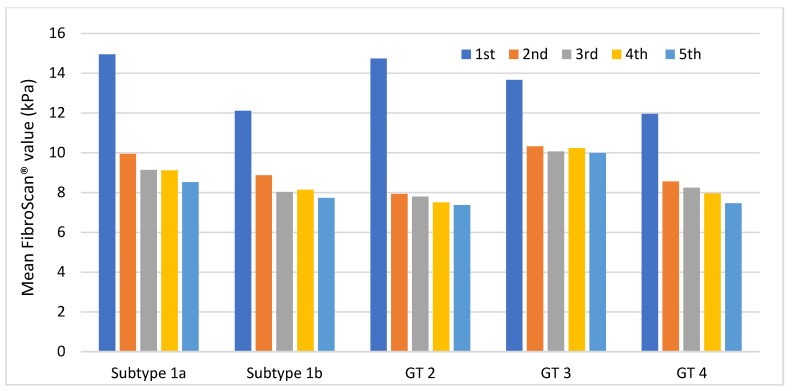
Trend of mean FibroScan^®^ values in each GT throughout the follow-up period. GT, viral genotype.

As shown in Figure 5, the highest mean values were observed in patients with subtype 1a and GT 2, being slightly higher in the former. Patients with GT 3 showed higher values than patients with subtype 1b and GT 4.

All subgroups showed a large decrease in their values at the second measurement and remained stable at subsequent measurements. Patients with GT 2 and GT 3 had the greatest and least decrease in their values, respectively. Further information regarding these results can be found in Table S3 (Supplementary Materials).

**Figure 6:**
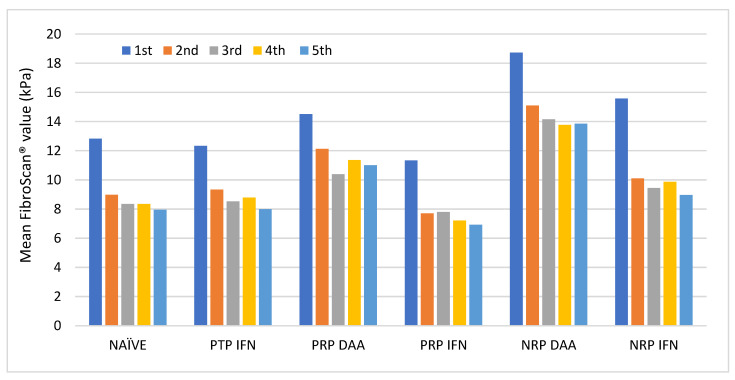
Trend of mean FibroScan^®^ values in each therapeutic regimen throughout the follow-up period. DAA, direct-acting antiviral; IFN, Interferon; NRP, no response to a previous therapy; PRP, partial response to a previous therapy; PTP, poor tolerance to a previous therapy.

As shown in Figure 6, the highest and lowest mean values were observed in patients with NRP DAA and PRP IFN, respectively. Patients with PRP DAA and NRP IFN showed close values, being slightly higher in the latter. Similarly, patients with PTP IFN and NAÏVE showed identical values.

Patients with NRP IFN showed the largest decrease in their values, although the other subgroups also decreased them at the second measurement and remained stable at subsequent measurements. Further information regarding these results can be found in Table S4 (Supplementary Materials).

In summary, the highest mean FibroScan^®^ values were observed in patients with “male gender”, “metabolic syndrome”, subtype 1a”, and “NRP DAA”.

#### 3.4.4. Complications

This subsection describes and analyzes mean FibroScan^®^ values based on HCV complications developed after completing the therapeutic regimen.

**Figure 7:**
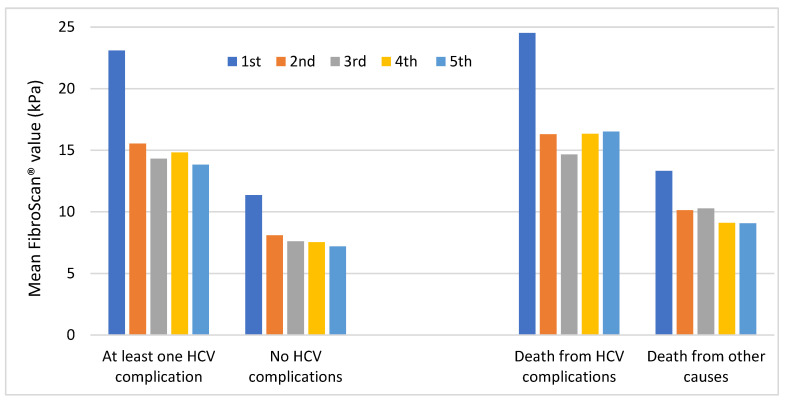
Trend of mean FibroScan^®^ values in patients with at least one HCV complication vs. patients without HCV complications throughout the follow-up period (**left**). Trend of mean FibroScan^®^ values in patients who died from HCV complications vs. patients who died from other causes throughout the follow-up period (**right**). HCV, hepatitis C virus.

As shown in Figure 7, patients who died from HCV complications had the highest mean values, although values in patients with at least one HCV complication were very similar. The mean values of each subgroup showed a great decrease at the second measurement and remained stable at subsequent measurements.

As the left side of this figure shows, mean values were much higher in patients with at least one HCV complication than in patients without. As the right side of this figure shows, mean values were much higher in patients who died from HCV complications than in patients who died from other causes. Further information regarding these results can be found in Table S5 (Supplementary Materials).

**Figure 8:**
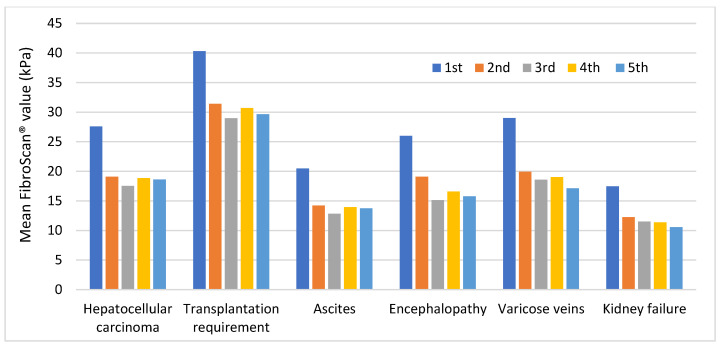
Trend of mean FibroScan^®^ values in each complication throughout the follow-up period.

As shown in Figure 8, the highest and lowest mean values were observed in patients with liver transplantation requirement and kidney failure, respectively. Patients with varicose veins, hepatocellular carcinoma, and encephalopathy showed similar values, being higher in the former.

All subgroups greatly decreased their values at the second measurement and remained stable at subsequent measurements. Patients with liver transplantation requirement and varicose veins had the largest decrease. Further information regarding these results can be found in Table S6 (Supplementary Materials).

In summary, the highest mean FibroScan^®^ values were observed in patients with “at least one HCV complication”, “death from HCV complications”, and “liver transplantation requirement”.

### 3.5. Distribution in Subgroups

**Figure 9:**
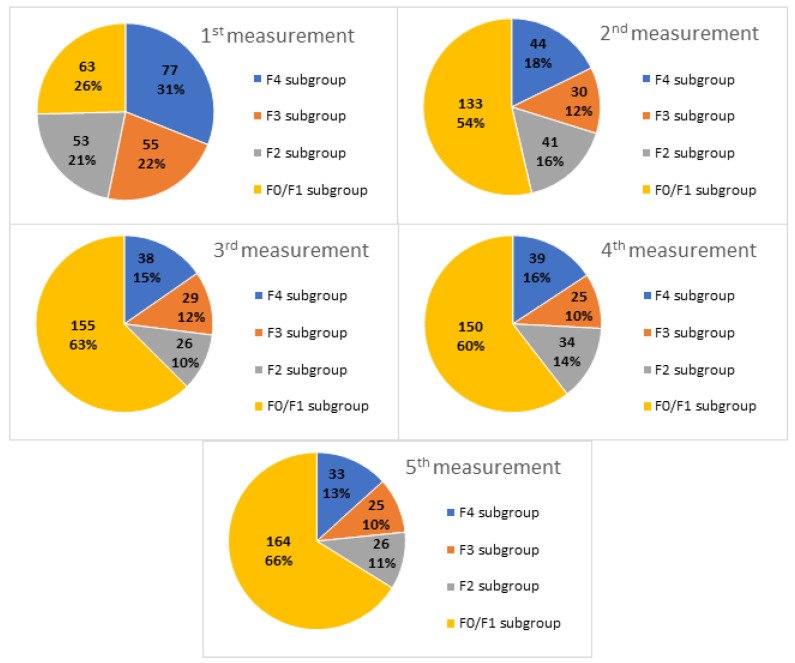
Graphic description of the distribution of patients in subgroups according to changes in the fibrotic stage throughout the follow-up period.

Variations in the distribution of patients into subgroups over time based on changes in the fibrotic stage are provided in Table 4 and Figure 9. Both show how the F0/F1 subgroup progressively increased throughout the follow-up period.

#### 3.5.1. F4 Subgroup

**Figure 10:**
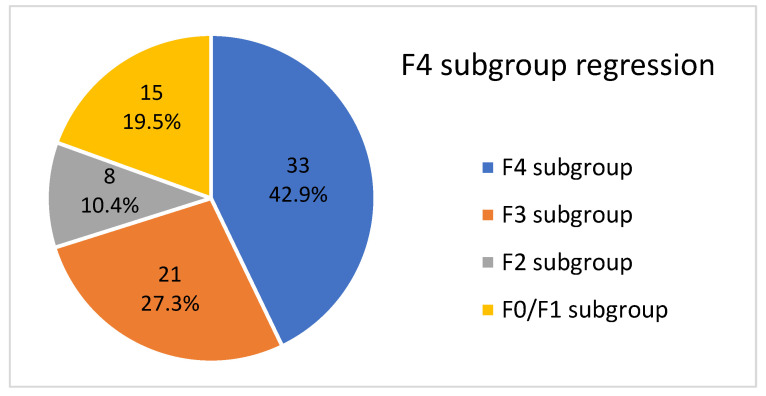
Graphic description of the fibrotic stage in F4 patients at the end of the follow-up period.

Of these 77 patients, 21 (27.3%) regressed to F3, 8 (10.4%) regressed to F2, and 15 (19.5%) regressed to F0/F1 at the end of the follow-up period. This is shown in Figure 10.

Therefore, a total of 44 patients (57.2%) abandoned their baseline cirrhotic stage, 17 (38.6%) achieved it after completing treatment at twelve weeks, 15 (34.1%) at one year, 2 (4.6%) at two years, and 10 (22.7%) at three years.

#### 3.5.2. F3, F2 and F0/F1 Subgroups

Of these 171 patients, none (0%) progressed to a higher fibrotic stage at the end of the follow-up period.

**Figure 11:**
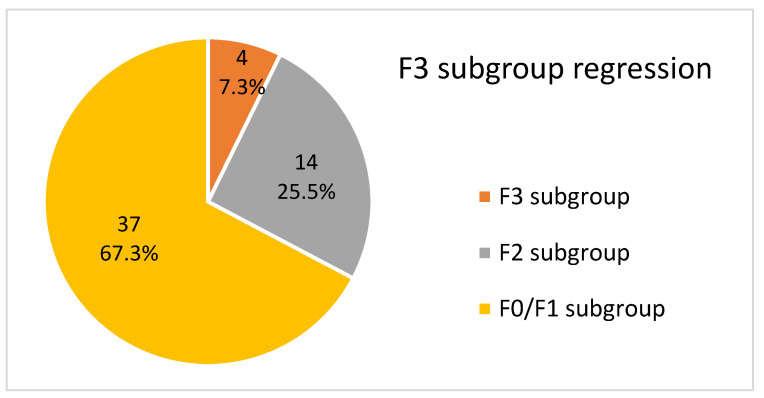
Graphic description of the fibrotic stage in F3 patients at the end of the follow-up period.

Of the 55 F3 patients, 51 (92.7%) regressed to a lower fibrotic stage, 14 (25.5%) regressed to F2, and 37 (67.3%) regressed to F0/F1. This is shown in Figure 11. Of these 51 patients, 30 (58.8%) achieved it after completing treatment at twelve weeks, 14 (27.5%) at one year, 5 (9.8%) at two years, and 2 (3.9%) at three years.

**Figure 12:**
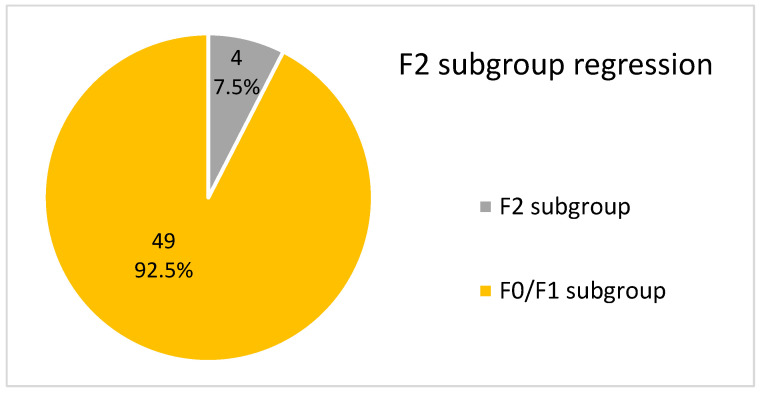
Graphic description of the fibrotic stage in F2 patients at the end of the follow-up period.

In addition, 49 of the 53 F2 patients (92.5%) regressed to F0/F1. This is shown in Figure 12. Of these 49 patients, 39 (79.5%) achieved it after completing treatment at twelve weeks, 6 (12.3%) at one year, and 4 (8.2%) at three years.

Overall, non-cirrhotic patients had a greater and faster regression to a lower fibrotic stage than cirrhotic patients.

### 3.6. Fibrotic Stage and Development of Hepatocellular Carcinoma

Due to its high prognostic importance, this section describes and analyzes the fibrotic stage in patients who developed hepatocellular carcinoma. Of these 13 patients, 12 (92.3%) were F4, and 1 (7.7%) was F3.

Of the 12 F4 patients, 10 (83.4%) maintained this fibrotic stage at the end of the follow-up period, 1 (8.3%) regressed to F3, and 1 (8.3%) regressed to F2. Their lowest baseline FibroScan^®^ value was 16.6 kPa.

The F3 patient had a baseline FibroScan^®^ value of 9.7 kPa, regressing to F1 at the end of the follow-up period.

Overall, no patient with an early fibrotic stage developed hepatocellular carcinoma.

## 4. Discussion

This work analyzed the therapeutic effectiveness of DAAs in terms of LFR, whose achievement reduces the incidence of HCV complications. As in other similar studies, high rates of LFR were observed [27]. Moreover, all subgroups decreased their mean FibroScan^®^ values. F4 patients were the least likely to achieve LFR and the most likely to develop HCV complications.

There was no recurrence in our series of F4 patients throughout the follow-up period, with an LFR rate close to 60%. A decreasing trend in mean FibroScan^®^ values was observed in more than 70% of patients who did not achieve this goal. This subgroup is at high risk of developing HCV complications, which were 35% in our series. The most important complication is hepatocellular carcinoma, which was slightly higher than 15% in our series [10]. No F4 patient with a baseline FibroScan^®^ value equal to or less than 16.5 kPa developed hepatocellular carcinoma. Based on current scientific evidence, six-monthly abdominal ultrasound check-ups are recommended in all F4 patients and all patients with ultrasound criteria for cirrhosis [28].

There was no recurrence in our series of F3 and F2 patients throughout the follow-up period. There were also no cases of progression to a higher fibrotic stage, with an LFR rate higher than 90%. Both subgroups are at intermediate risk of developing HCV complications (medium–high in F3 patients and medium–low in F2 patients), which were less than 10% in our series. The appearance of a case of hepatocellular carcinoma with 9.7 kPa as the baseline FibroScan^®^ value must be highlighted.

There was no recurrence in our series of F0/F1 patients throughout the follow-up period. There were also no cases of progression to a higher fibrotic stage, with a decreasing trend in mean FibroScan^®^ values in more than 95% of cases. This subgroup is at low risk of developing HCV complications, which were less than 5% in our series.

Considering the findings of this work, six-monthly abdominal ultrasound check-ups could be considered in all patients who have developed HCV complications, regardless of the baseline fibrotic stage. Then, annual abdominal ultrasound check-ups could be considered in F3 patients without ultrasound criteria for cirrhosis who have not developed HCV complications after two years and whose fibrotic stage is not F0/F1.

Considering the findings of this work, completion of follow-up could be considered in F2 patients without ultrasound criteria for cirrhosis who have not developed HCV complications after two years, and whose fibrotic stage is F0/F1. Then, completion of follow-up could be considered in F0/F1 patients without ultrasound criteria for cirrhosis who have not developed HCV complications after one year.

In comparison to similar studies, patients in our cohort had fewer side effects, which were mild and infrequent. Then, there was no discontinuation or change in the therapeutic regimen [29].

Compared to similar studies, patients in our cohort showed lower rates of HCV mortality and complications, with lower development of hepatocellular carcinoma (5.2% vs. 5.52%). As in most studies, a higher prevalence of men and patients with GT 1 was also observed [30].

Compared to studies that suggest a possible association between GT 3 and faster fibrotic progression, GT 3 patients in our cohort did not have the highest fibrotic values. However, they decreased their fibrotic values less than the other genotypes [31].

The main limitations of this work were the typical biases of a retrospective study. We can highlight the losses to follow-up and the inability to perform adequate comparisons in subgroups with few patients. In addition, FibroScan^®^ has high interobserver variability so that the liver stiffness value could change depending on the physician performing the measurement [23,24].

## 5. Conclusions

In this cohort study, composed of patients with chronic HCV infection who achieved SVR for at least five years, DAA therapy was highly effective in achieving LFR and decreasing mean FibroScan^®^ values in all subgroups. Patients in the F4 subgroup showed the lowest LFR rates and the highest development of HCV complications.

## Figures and Tables

**Table 1:** Distribution of patients in subgroups according to the baseline fibrotic value.

METAVIR Scale	Liver Fibrosis (kPa)
F0/F1 subgroup	≤7.4
F2 subgroup	7.5–9.4
F3 subgroup	9.5–12.4
F4 subgroup	≥12.5

kPa, kilopascals.

**Table 2:** Quantitative description of each baseline characteristic.

Parameter	HCV Infected (*n* = 248)	*p* =
Males/females, *n* (%)	175 (70.6)/73 (29.4)	0.011
Age (years) mean (SD)	54.6 (10.2)	0.000
High active alcohol use, *n* (%)	24 (9.7)	0.007
Mild liver steatosis, *n* (%)	33 (13.3)	0.087
Metabolic syndrome, *n* (%)	24 (9.7)	0.042
HIV, *n* (%)	30 (12.1)	0.065
NAÏVE, *n* (%)	159 (64.1)	0.029
PTP IFN, *n* (%)	8 (3.2)	
PRP DAA, *n* (%)	11 (4.5)	
NRP DAA, *n* (%)	6 (2.4)	
PRP IFN, *n* (%)	28 (11.3)	
NRP IFN, *n* (%)	36 (14.5)	
Subtype 1a	80 (32.2)	0.018
Subtype 1b	108 (43.6)	
GT 2	3 (1.3)	
GT 3	36 (14.5)	
GT 4	21 (8.4)	
F4 subgroup, *n* (%)	77 (31.0)	0.667
F3 subgroup, *n* (%)	55 (22.2)	
F2 subgroup, *n* (%)	53 (21.4)	
F0/F1 subgroup, *n* (%)	63 (25.4)	

DAA, direct-acting antiviral; GT, viral genotype; HCV, hepatitis C virus; HIV, human immunodeficiency virus; IFN, Interferon; NRP, no response to a previous therapy; PRP, partial response to a previous therapy; PTP, poor tolerance to a previous therapy; SD, standard deviation.

**Table 3:** Quantitative description of each complication developed according to the baseline fibrotic stage.

	F4 Subgroup (*n* = 77)	F3 Subgroup (*n* = 55)	F2 Subgroup (*n* = 53)	F0/F1 Subgroup (*n* = 63)
At least one HCV complication	27 (35.1%)	5 (9.1%)	6 (11.3%)	2 (3.2%)
Death from HCV complications	4 (5.2%)	1 (1.8%)	0 (0%)	0 (0%)
Hepatocellular carcinoma	12 (15.6%)	1 (1.8%)	0 (0%)	0 (0%)
Ascites	9 (11.7%)	3 (5.4%)	2 (3.8%)	0 (0%)
Varicose veins	17 (22.1%)	1 (1.8%)	1 (1.9%)	0 (0%)
Encephalopathy	4 (5.2%)	1 (1.8%)	0 (0%)	0 (0%)
Kidney failure	5 (6.5%)	0 (0%)	4 (7.6%)	2 (3.2%)
Liver transplantation requirement	6 (7.8%)	0 (0%)	0 (0%)	0 (0%)

HCV, hepatitis C virus.

**Table 4:** Quantitative description of the distribution of patients in subgroups according to changes in the fibrotic stage throughout the follow-up period.

Measurements	F4 Subgroup	F3 Subgroup	F2 Subgroup	F0/F1 Subgroup
1st	77 (31.0%)	55 (22.2%)	53 (21.4%)	63 (25.4%)
2nd	44 (17.8%)	30 (12.1%)	41 (16.5%)	133 (53.6%)
3rd	38 (15.3%)	29 (11.7%)	26 (10.5%)	155 (62.5%)
4th	39 (15.7%)	25 (10.1%)	34 (13.7%)	150 (60.5%)
5th	33 (13.3%)	25 (10.1%)	26 (10.5%)	164 (66.1%)

## Data Availability

The data presented in this study are available on request from the corresponding author.

## Supplementary Materials

The following supporting information can be downloaded at: https://www.mdpi.com/article/10.3390/medicina59040814/s1, Table S1: Quantitative description of mean FibroScan^®^ values in men vs. women throughout the follow-up period. Table S2: Quantitative description of mean FibroScan^®^ values in patients with high active alcohol use, mild liver steatosis, and metabolic syndrome throughout the follow-up period. Table S3: Quantitative description of mean FibroScan^®^ values in each GT throughout the follow-up period. Table S4: Quantitative description of mean FibroScan^®^ values in each therapeutic regimen throughout the follow-up period. Table S5: Quantitative description of mean FibroScan^®^ values in patients with at least one HCV complication vs. patients without HCV complications throughout the follow-up period (left side). Quantitative description of mean FibroScan^®^ values in patients who died from HCV complications vs. patients who died from other causes throughout the follow-up period (right side). Table S6: Quantitative description of mean FibroScan^®^ values in each complication throughout the follow-up period.
