# Supplementary material for: Impact of Direct-Acting Antiviral Therapy on Liver Fibrosis Regression among People with Chronic HCV Infection: Results from a Real-Life Cohort in Patients Who Achieved Sustained Virological Response
Source: Medicina (Kaunas). 2023 Apr 21;59(4):814. doi: 10.3390/medicina59040814 (PMC10141631; doi:10.3390/medicina59040814)
Supplement: Supplementary file 1 [file medicina-59-00814-s001.zip › medicina-2289190-supplementary.pdf]

medicina MDPI

Table S1. Quantitative description of mean FibroScan® values in men vs. women patients throughout the follow-up period.

	Mean FibroScan® value (kPa)	
**Measurements**	**Men**	**Women**
1st	13.645	12.359
2nd	9.465	8.484
3rd	9.124	7.667
4th	9.009	8.036
5th	8.582	7.524

Table S2. Quantitative description of mean FibroScan® values in patients with high active alcohol use, mild liver steatosis and metabolic syndrome throughout the follow-up period.

	Mean FibroScan® value (kPa)		
**Measurements**	**High active alcohol use**	**Mild liver steatosis**	**Metabolic syndrome**
1st	14.075	14.542	17.171
2nd	10.712	10.564	12.808
3rd	9.162	9.306	10.662
4th	9.546	9.467	10.883
5th	8.979	9.067	10.429

Table S3. Quantitative description of mean FibroScan® values in each GT throughout the follow-up period.

	Mean FibroScan® value (kPa)				
**Measurements**	**GT 1A**	**GT 1B**	**GT 2**	**GT 3**	**GT 4**
1st	14.953	12.106	14.733	13.668	11.957
2nd	9.941	8.867	7.933	10.321	8.552
3rd	9.141	8.026	7.8	10.068	8.243
4th	9.112	8.137	7.5	10.232	7.957
5th	8.525	7.728	7.367	9.982	7.461

Table S4. Quantitative description of mean FibroScan® values in each therapeutic regimen throughout the follow-up period.

	Mean FibroScan® value (kPa)					
**Measurements**	**NAÏVE**	**PTP IFN**	**PRP DAA**	**PRP IFN**	**NRP DAA**	**NRP IFN**
1st	12.82	12.338	14.509	11.336	18.733	15.583
2nd	8.975	9.337	12.127	7.696	15.1	10.1
3rd	8.352	8.525	10.382	7.796	14.15	9.442
4th	8.342	8.788	11.355	7.211	13.767	9.864
5th	7.952	7.987	11	6.925	13.85	8.961

Table S5. Quantitative description of mean FibroScan® values in patients with at least one HCV complication vs. patients without HCV complications throughout the follow-up period (left side). Quantitative description of mean FibroScan® values in patients who died from HCV complications vs. patients who died from other causes throughout the follow-up period (right side).

	Mean FibroScan® value (kPa)			
**Measurements**	**At least one HCV complication**	**No HCV complications**	**Death from HCV complications**	**Death from other causes**
1st	23.095	11.364	24.52	13.333
2nd	15.535	8.094	16.3	10.133
3rd	14.312	7.6	14.66	10.278
4th	14.82	7.54	16.34	9.111
5th	13.828	7.191	16.52	9.078

Table S6. Quantitative description of mean FibroScan® values in each complication throughout the follow-up period.

	Mean FibroScan® value (kPa)					
**Measurements**	**Hepatocellular carcinoma**	**Liver transplantation requirement**	**Ascites**	**Encephalopathy**	**Varicose veins**	**Kidney failure**
1st	27.582	40.333	20.486	25.98	29.021	17.464
2nd	19.1	31.417	14.214	19.08	19.947	12.245
3rd	17.527	28.983	12.836	15.14	18.568	11.5
4th	18.845	30.7	13.95	16.6	19.026	11.364
5th	18.6	29.65	13.743	15.76	17.132	10.564
